# Variations in Stress Ulcer Prophylaxis Practices Among Intensive Care Units in Makkah City Hospitals, Kingdom of Saudi Arabia

**DOI:** 10.7759/cureus.87945

**Published:** 2025-07-14

**Authors:** Jeehad M Felemban, Ammar Z Faloudah, Abdullah A Allahyani, Ahmed Y Shebly, Mohammed A Aljehani, Mohammed A Alosaime, Mohammed A Almalki, Manar M Alslymi, Ali S Almatrafi, Mohannad M AbuRageila

**Affiliations:** 1 Adult Critical Care Medicine, Security Forces Hospital, Makkah, SAU; 2 Adult Critical Care Medicine, King Abdullah Medical City, Makkah, SAU; 3 Respiratory Therapy, Security Forces Hospital, Makkah, SAU; 4 Department of Medicine, College of Medicine, Umm Al-Qura University, Makkah, SAU; 5 Pulmonary and Adult Critical Care Medicine, Security Forces Hospital, Makkah, SAU

**Keywords:** critically ill patients, intensive care unit, practice variation, stress-related mucosal disease, stress ulcer prophylaxis

## Abstract

Introduction

Stress-related mucosal disease (SRMD) encompasses a spectrum of gastric mucosal injuries, ranging from superficial erosions to deep ulcerations, typically occurring in critically ill patients due to mucosal ischemia and impaired protective mechanisms. It is a frequent and serious complication among patients in intensive care units (ICUs), with stress ulcer prophylaxis (SUP) widely used to reduce the risk of gastrointestinal (GI) bleeding. However, inappropriate use of SUP can expose patients to avoidable risks, including ventilator-associated pneumonia and *Clostridium difficile* infections. Despite the availability of international guidelines, variations in SUP practices persist across healthcare settings. This study aimed to identify the current local practice of SUP use and to evaluate awareness of international guidelines and associated challenges among ICU physicians in Makkah City hospitals, Saudi Arabia.

Methods

This cross-sectional study was conducted from July to September 2024 among 92 ICU physicians working in adult intensive care units across Makkah city hospitals, Saudi Arabia. Physicians with more than three months of ICU experience who were involved in stress ulcer prophylaxis management were included, while those with less experience or working exclusively in pediatric/neonatal ICUs were excluded. Data were collected via a validated electronic questionnaire assessing SUP practices and awareness of related guidelines and complications. Statistical analysis was performed using IBM Corp. Released 2017. IBM SPSS Statistics for Windows, Version 26.0. Armonk, NY: IBM Corp. Ethical approval and informed consent were obtained.

Results

A total of 92 ICU physicians participated in the survey. Most respondents, 83 (90%), reported familiarity with stress ulcer prophylaxis (SUP) guidelines. Timing and indications for initiating SUP varied, with 45 physicians (48%) initiating it based on major risk factors, while 28 (31%) supported its use even in the absence of clear risk factors. Sixty-two physicians (67%) endorsed universal SUP use, whereas 30 (33%) preferred selective administration. Intravenous proton pump inhibitors (PPIs) were the preferred agent among 73 participants (79.1%). Reported barriers to consistent SUP practice included the lack of daily medication reviews, cited by 59 participants (63.7%), and limited pharmacist support, noted by 36 participants (39.6%). Most participants, 57 (48%), indicated that SUP decisions were made individually by ICU physicians rather than through standardized protocols.

Conclusion

Despite high awareness of SUP guidelines, considerable variability in practice persists among ICU physicians in Makkah City. Standardized protocols, enhanced pharmacist involvement, and targeted educational initiatives are essential to promote evidence-based SUP use and minimize associated risks.

## Introduction

Stress-related mucosal disease (SRMD) is a well-documented and frequent complication, particularly among patients in intensive care units (ICUs) [1,2]. Some studies estimate that 75% to 100% of ICU patients develop gastric lesions, often within the first day of admission [1-3]. These lesions can range from superficial erosions to deeper ulcerations [1,2]. The pathogenesis of SRMD involves multiple mechanisms, most notably splanchnic hypoperfusion. This condition results in reduced mucosal blood flow, decreased bicarbonate secretion, impaired gastrointestinal motility, and increased acid back-diffusion, collectively leading to mucosal barrier breakdown [3,4]. Several risk factors contribute to the development of SRMD, including mechanical ventilation, coagulopathy (defined as an INR >1.5, platelet count <50,000, or partial thromboplastin time more than twice the control value), head trauma, hepatic failure, renal failure, and thermal injuries [2,3,5]. ICU patients are particularly susceptible to stress ulcers due to the combination of preexisting chronic conditions and the severity of acute illnesses that necessitate intensive care admission [6,7]. Stress ulcer prophylaxis (SUP) is therefore considered an essential component in the management of critically ill patients. Although the incidence of SRMD and associated gastrointestinal bleeding (GIB) has significantly declined over the past decades, stress-related bleeding still poses a serious risk, potentially leading to increased mortality, prolonged hospital stays, and greater healthcare costs. Despite gastric mucosal lesions being highly prevalent among ICU patients, the incidence of stress-related GIB is much lower, as current data estimate the incidence to range between 2% and 5% [4,7,8,9]. However, SUP is frequently overprescribed, often without proper clinical justification. This inappropriate use may expose patients to avoidable adverse outcomes, such as an increased risk of ventilator-associated pneumonia, *Clostridium difficile* infections, and myocardial ischemia [9,10]. While the evidence linking SUP to these adverse effects varies in strength, the potential for harm remains significant. Additionally, the overall quality of evidence supporting SUP use is limited [5]. Nonetheless, several studies have shown that SUP, when appropriately prescribed to high-risk ICU patients, can effectively reduce the incidence of GIB and shorten hospital stays [5,7,9]. In recent years, anecdotal observations have indicated that SUP is frequently prescribed for nearly all hospitalized patients, a practice that is inconsistent with current international guidelines [11,12]. Therefore, this study aims to identify the current local practices of stress ulcer prophylaxis (SUP) use in ICUs at Makkah City hospitals, assess physicians’ knowledge of relevant international guidelines, and evaluate whether this knowledge is reflected in systematic, protocol-driven practice.

## Materials and methods

This is a cross-sectional study that aims to provide descriptive data regarding the current local practice of using stress ulcer prophylaxis in intensive care units at Makkah city hospitals and also physicians' knowledge of the current international guidelines and recommendations of SUP in ICU patients [11,12]. The study was conducted over two months, from July 21, 2024, to September 20, 2024. The sample size was determined using an online sample size calculator (Raosoft, Seattle, US) [19]. The target population included 120 intensive care unit (ICU) physicians working in seven hospitals across Makkah, Saudi Arabia. With a confidence level of 95%, a margin of error of 5%, and a response distribution of 50%, the calculated required sample size was 92 participants, which was deemed sufficient to achieve statistical power and representativeness for the study population. Random generator software was used for randomization, and a stratified random sampling approach was utilized, with stratification based on hospital affiliation. The calculated sample size was distributed equally across the seven participating government hospitals in Makkah City to ensure geographic and institutional representation. No further stratification was applied based on physician rank or ICU type. This study targeted physicians currently practicing in adult intensive care units within government hospitals in Makkah City, Kingdom of Saudi Arabia. Eligible participants were those with a minimum of three months’ experience in ICU practice and actively engaged in the prescribing or clinical management of stress ulcer prophylaxis (SUP). The sample comprised ICU clinicians across different professional ranks, including specialists, residents, and consultants, to capture diverse perspectives on SUP practices. Physicians with less than three months of ICU experience, as well as those assigned exclusively to pediatric or neonatal ICUs, were excluded. Private-sector hospitals were not included in the sampling frame. The primary objectives of the study were to investigate current practices of ICU physicians regarding SUP, specifically initiation practices, preferred pharmacologic agents, and local prescribing patterns. Additionally, the study aimed to assess physicians’ awareness of international SUP guidelines, the protective role of early enteral feeding, and complications associated with inappropriate SUP use, including ventilator-associated pneumonia and *Clostridium difficile* infection. Data were collected using a self-administered electronic questionnaire. The questionnaire was adapted from a previously published and validated instrument used in a similar study assessing ICU clinical practices related to stress ulcer prophylaxis [13]. Minor modifications were made to the original instrument, including the addition of new items, to ensure alignment with the local healthcare setting and clinical protocols in Makkah, Saudi Arabia. The final version was reviewed by domain experts to ensure content validity. A pilot test was conducted with a sample of 12 ICU physicians who were not included in the final study. Internal consistency of the questionnaire was assessed using Cronbach’s alpha, which was 0.81, indicating good reliability. Based on participant feedback, minor adjustments were made to improve the clarity and structure of the questionnaire before its administration in the main study. The questionnaire consisted of three parts. The first part focused on demographic information. The second part assessed participants' routine practices regarding the initiation of SUP. The third part evaluated participants’ knowledge of gastrointestinal bleeding risk factors, guideline recommendations of SUP, and potential complications linked to SUP overuse. Data were analyzed using IBM Corp. Released 2017. IBM SPSS Statistics for Windows, Version 26.0. Armonk, NY: IBM Corp. Descriptive statistics, including frequencies and percentages, were used for categorical variables, while means and standard deviations were used for continuous variables. Research committee approval was obtained from the relevant research committee, and both verbal and written consent were secured from all participants after explaining the nature and purpose of the study.

## Results

**Figure 1:**
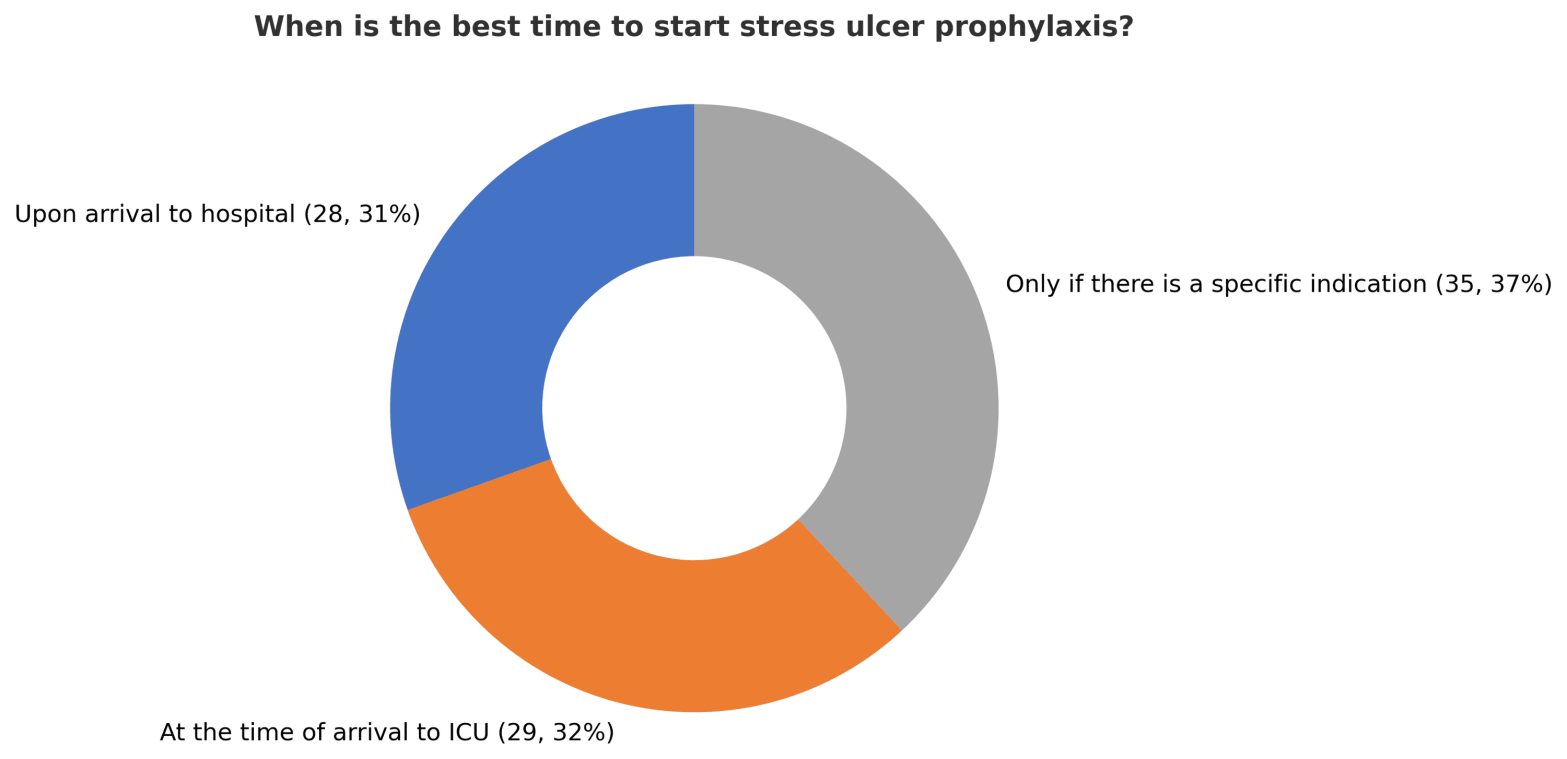
Timing preferences for initiating stress ulcer prophylaxis among ICU clinicians (N = 92)

**Figure 2:**
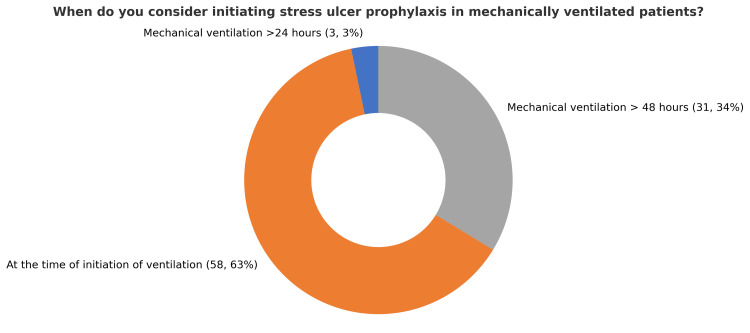
Timing of stress ulcer prophylaxis initiation in mechanically ventilated patients (N = 92)

**Figure 3:**
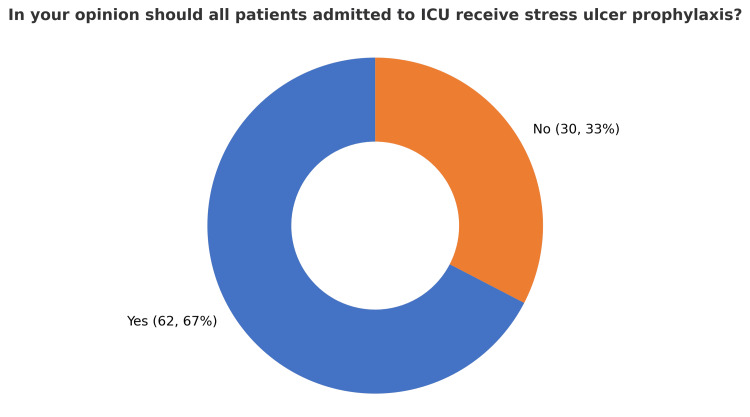
ICU physicians’ opinions on universal stress ulcer prophylaxis (N = 92)

**Figure 4:**
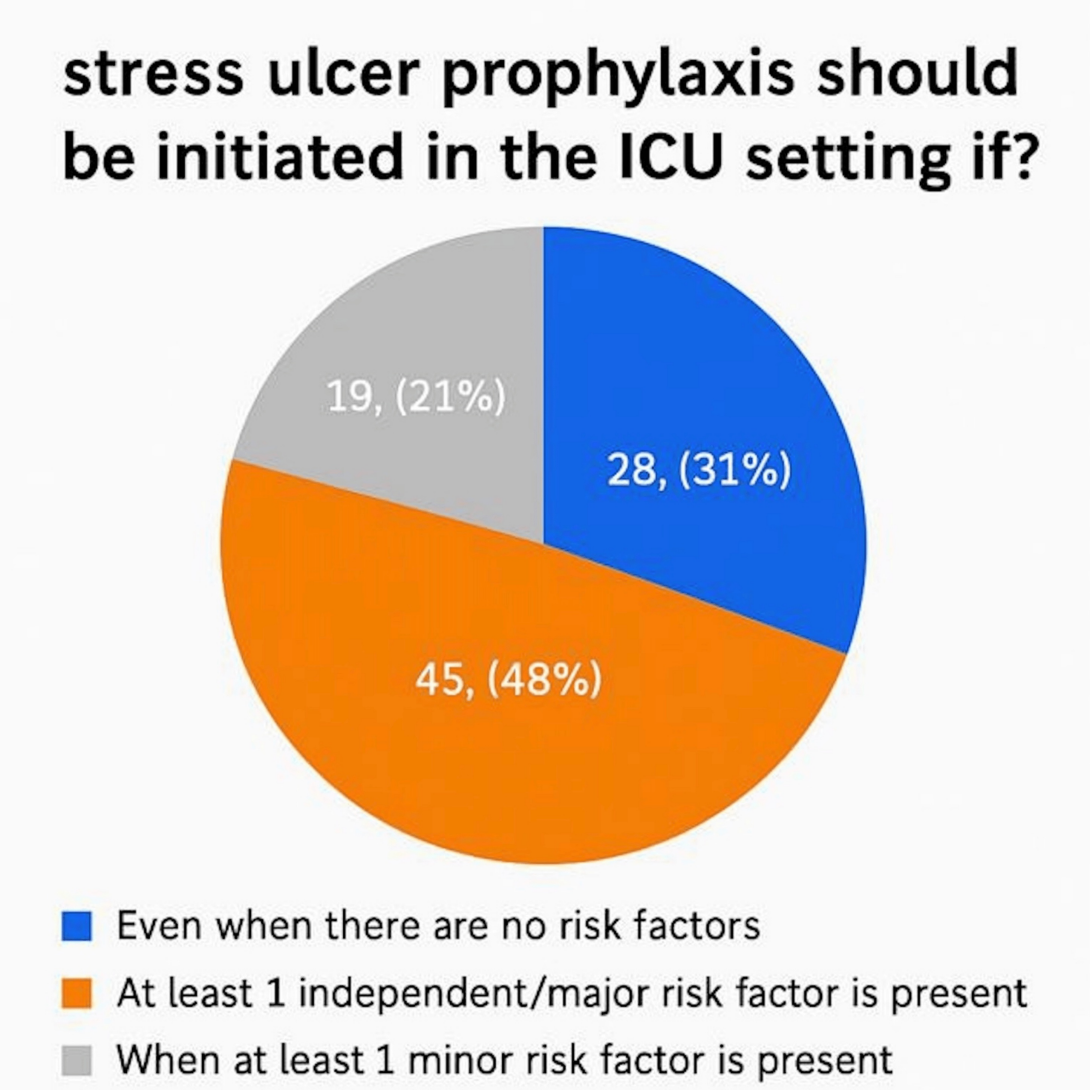
Physicians’ thresholds for starting stress ulcer prophylaxis in ICU patients (N = 92)

**Figure 5:**
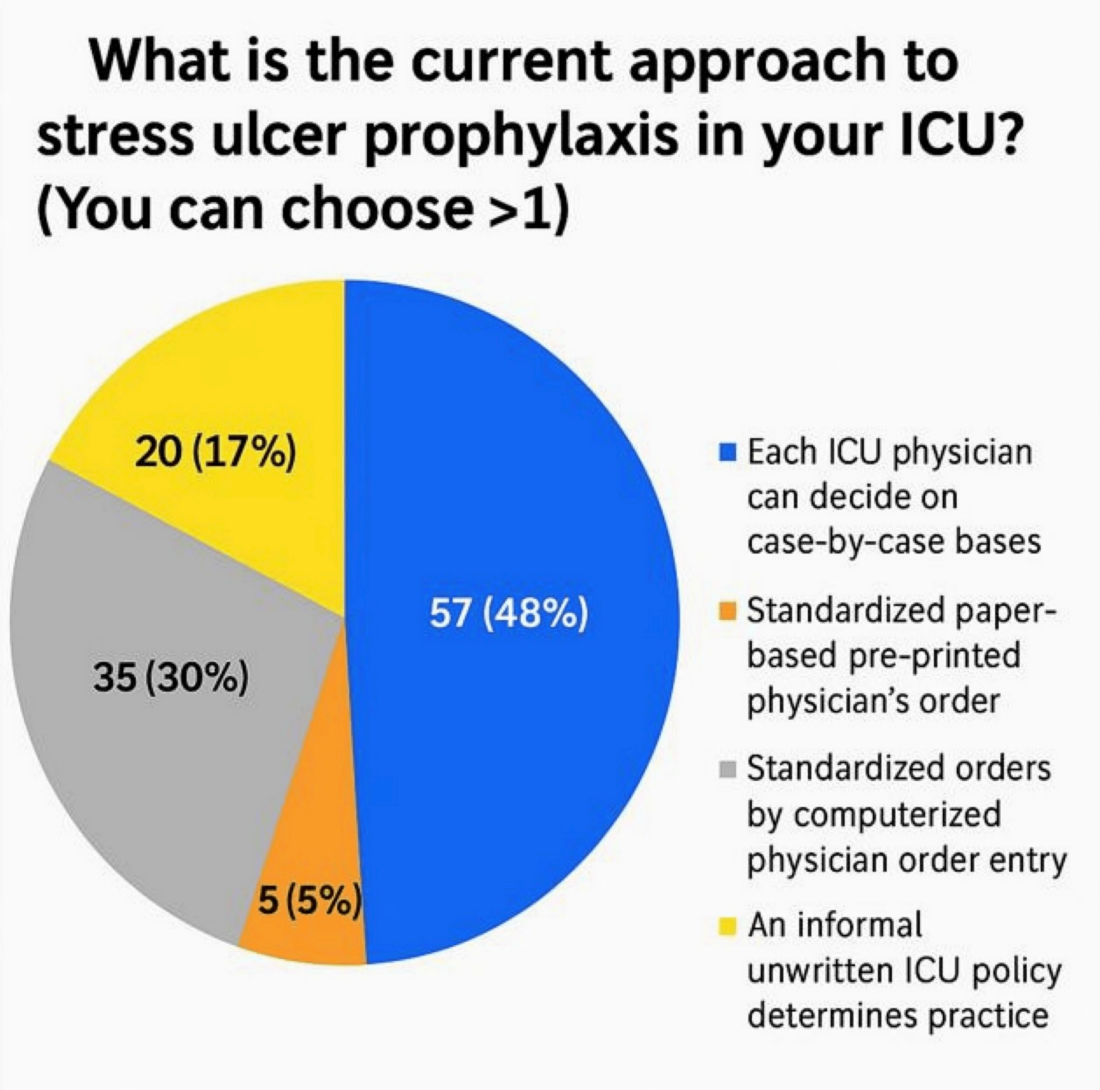
Approaches to stress ulcer prophylaxis in ICUs (N = 92)

A total of 92 ICU physicians across seven governmental hospitals in Makkah city participated in the survey, which assessed their practices, knowledge, and attitudes toward stress ulcer prophylaxis (SUP). The participants included 59 specialists (64.8%), 22 residents (24.2%), and 10 consultants (11%). This diverse representation reflects varying levels of expertise and responsibility within ICU settings. Awareness of stress ulcer prophylaxis guidelines was high, with 83 participants (90%) reporting familiarity with standard protocols. In contrast, nine participants (10%) indicated they were not aware of any existing guidelines. Among participants familiar with SUP guidelines, 36 individuals (44%) reported referencing versions published between 2015 and 2020, representing the highest proportion compared to other timeframes. Opinions regarding the best timing of SUP initiation varied. A total of 35 participants (37%) believed SUP should be initiated only for specific indications, while 29 (32%) participants recommended starting it upon ICU arrival. Another 29 participants (31%) favored initiating SUP at hospital admission, highlighting the diversity of clinical practices (Figure 1). Furthermore, in relation to the timing of stress ulcer prophylaxis initiation, 58 (63%) of respondents recommended starting therapy at the time of mechanical ventilation, whereas 31 (34%) suggested initiating it 48 hours after ICU admission. A small proportion of three participants (3%) advocated for a 24-hour delay prior to starting SUP (Figure 2). Additionally, 62 participants (67%) supported a universal approach, administering SUP to all ICU patients, whereas 30 (33%) favored a selective strategy based on individual risk factors (Figure 3). A majority of respondents, 83 (90%), agreed that early enteral tube feeding within 48 hours of ICU admission is protective against stress-related mucosal disease (SRMD). Additionally, 80 (87%) believed that acid-suppressive therapy reduces the risk of gastrointestinal (GI) bleeding; however, 12 (13%) disagreed. Participants demonstrated varied approaches regarding the initiation of stress ulcer prophylaxis (SUP) in relation to major and minor risk factors. The largest proportion, 45 participants (48%), indicated that SUP should be initiated when at least one independent or major risk factor is present. Meanwhile, 19 (21%) supported initiation based on a single minor risk factor, and notably, 28 (31%) advocated for initiating prophylaxis even in the absence of identifiable risk factors (Figure 4). Regarding risk factors for gastrointestinal (GI) bleeding, the most important predictors identified by respondents included a platelet count <50,000, reported by 66 participants (71.4%), and an INR >1.5, reported by 62 participants (67%). Moreover, glucocorticoid therapy (≥250 mg/day) and respiratory failure were also frequently cited as minor risk factors, each identified by 41 participants (45.1%). The majority of participants preferred proton pump inhibitors (PPIs) for high-risk patients, with 73 participants (79.1%) selecting intravenous PPIs as the drug of choice. Enteral PPIs were favored by 15 participants (16.5%), while only four participants (4.4%) chose sucralfate. When asked about the adverse effects of PPIs, ventilator-associated pneumonia (VAP) was reported by 58 participants (62.6%), and *Clostridium difficile* infection was selected by 52 participants (56%). Reported barriers to consistent adherence with SUP protocols included the lack of routine daily medication reviews, cited by 59 participants (63.7%), and limited clinical pharmacist support, cited by 36 participants (39.6%). Additional challenges identified were high patient workloads, noted by 27 participants (29.7%), and knowledge gaps among staff, reported by 45 participants (48.4%). Opinions regarding the appropriate timing for discontinuation of stress ulcer prophylaxis varied among respondents: A total of 39 participants (42.9%) recommended stopping SUP once the initial indication was resolved, while 27 participants (29.7%) favored continuing it throughout the hospital stay, and 25 participants (27.5%) suggested continuing it throughout the ICU stay. When asked about current approaches to stress ulcer prophylaxis in ICUs, participants indicated a predominant reliance on individualized decision-making. The majority, 57 participants (48%), reported that each ICU physician determines SUP use on a case-by-case basis. Additionally, 35 (30%) cited the use of standardized orders through computerized physician order entry (CPOE) systems. An informal, unwritten ICU policy was reported by 20 (17%) of respondents, while only five (5%) noted the use of standardized, paper-based pre-printed orders (Figure 5).

## Discussion

Stress ulcer prophylaxis (SUP) has traditionally been considered an important intervention in the management of intensive care unit (ICU) patients, primarily aimed at preventing stress-related mucosal disease (SRMD) and associated gastrointestinal (GI) bleeding. While SUP is widely adopted, its use has been increasingly debated in literature and by international guidelines due to concerns about overutilization, a lack of demonstrated mortality benefit, and the increased risk of nosocomial infections [5,11]. This study sought to examine ICU clinicians' practices, knowledge, and attitudes toward SUP, highlighting notable variations in practice and adherence to established clinical guidelines. The findings were compared with existing literature to provide context for their implications. Our study found a high level of awareness among ICU clinicians about SUP guidelines, with 90.1% of participants indicating familiarity with these protocols [11,12]. Despite this, 9.9% of ICUs lacked formal SUP protocols, suggesting inconsistency in clinical practice. This finding is consistent with the literature, which shows that while guidelines are in place, adherence often varies due to differences in institutional policies and physician preferences [11]. Similar trends were observed in a Saudi-based cross-sectional study by Alhujilan et al. [14], which assessed adherence to SUP guidelines. Their findings revealed suboptimal compliance, particularly in prescribing SUP for low-risk patients and failing to discontinue it when indications are resolved. The study also found that many clinicians, especially junior staff, were unaware of national guidelines. Our findings also reflect a predominant reliance on individualized decision-making in the initiation and management of SUP, rather than standardized protocols. This observation is consistent with a prior audit of SUP practices by Gupta et al. [13], which similarly reported considerable variability in ICU clinicians’ approaches, largely influenced by individual physician preference rather than institutional guidelines. A 2020 BMJ meta-analysis noted that inconsistent adherence to guidelines frequently leads to unnecessary SUP prescriptions in low-risk patients, increasing the risk of complications such as pneumonia and *Clostridium difficile* infection [15]. Clinicians' opinions on the timing of SUP initiation were diverse. Thirty-seven percent (37%) recommended SUP only for specific high-risk patients, while 32% suggested initiating it upon ICU admission, and 31% advocated starting it at hospital admission. This reflects ongoing debates in clinical practice about the appropriate indications for SUP. According to the ASHP guidelines and Bardou et al. (2015), SUP should be initiated in critically ill patients who present with major risk factors such as mechanical ventilation for more than 48 hours and coagulopathy (e.g., platelet count <50,000/mm³, INR >1.5, or aPTT >2× control). Additional risk factors may include a history of gastrointestinal bleeding, traumatic brain injury, burns, or sepsis. Aligning clinical practice with these criteria may help reduce unnecessary SUP use in low-risk patients [2,11,12]. Literature suggests that SUP should be reserved for high-risk patients, as the incidence of clinically significant GI bleeding in ICU settings is relatively low (0.5%-8.5%), particularly among patients without identified risk factors [2,4]. In a study by Krag et al. (2018), clinically significant GI bleeding occurred in only 2.6% of ICU patients, underscoring that most ICU patients do not require routine SUP [5]. Our study confirmed that major risk factors, such as mechanical ventilation for more than 48 hours and coagulopathy (platelet count <50,000 or INR >1.5), were identified by 71.4% and 67% of participants, respectively, as crucial indicators for initiating SUP. These risk factors align with those outlined in guidelines by Cook et al. (1999), Bardou et al. (2015), and the ASHP [16]. Regarding pharmacologic choices for SUP, our study found that 79.1% of clinicians preferred intravenous proton pump inhibitors (PPIs), while 16.5% used enteral PPIs, and only 4.4% opted for sucralfate. This preference is consistent with recent trials and meta-analyses, which show that PPIs are the most effective agents for acid suppression in ICU patients [13]. However, concerns about their potential adverse effects, particularly the increased risk of ventilator-associated pneumonia (VAP) and *Clostridium difficile* infection, remain. Our study revealed that 62.6% of participants were concerned about VAP, and 56% cited *Clostridium difficile* infection as a potential complication. A study by Krag et al. (2018) found no mortality benefit from PPI use over placebo but observed an increase in nosocomial pneumonia and *Clostridium difficile *infections [5]. The 2020 BMJ meta-analysis similarly found a 1.39-fold increase in pneumonia risk associated with PPIs (OR 1.39, 95% CI: 0.98-2.10) [15]. These findings suggest that routine PPI use in all ICU patients may not be warranted, given the potential infection risks. In terms of SUP discontinuation, 42.9% of clinicians in our study recommended stopping SUP once the indication for its use had resolved, while 29.7% favored continuing it throughout the hospital stay, and 27.5% suggested continuing it throughout the ICU stay. This prolonged use contradicts evidence suggesting that discontinuing SUP after resolving risk factors does not increase GI bleeding rates [3,4]. Studies by El-Kersh et al. (2018) and Palm et al. (2016) found that enteral nutrition alone is sufficient for stress ulcer prevention in many ICU patients, with PPIs offering no added benefit [17,18]. Pharmacist-led interventions have been shown to reduce inappropriate SUP use by 67.1%, resulting in significant cost savings, further supporting the need for better stewardship in SUP practices [15]. This study has several strengths. First, it included a diverse sample of ICU clinicians, including specialists, residents, and consultants, providing a broad perspective on SUP practices. Second, the findings largely align with existing guidelines on SUP risk stratification and pharmacologic choices, reinforcing their validity. Third, we contextualized our results with data from large-scale trials and meta-analyses, ensuring that the findings are clinically relevant. However, the study also has some limitations. The reliance on self-reported data introduces the possibility of recall bias or overestimation of adherence to guidelines. Additionally, the study was conducted within a specific geographical area, which may limit the generalizability of the results to other ICU settings. Furthermore, the study did not assess patient-level clinical outcomes, such as rates of gastrointestinal bleeding or *Clostridium difficile* infection, which could have provided more direct insights into the practical consequences of different SUP practices. In light of the findings and observed variations in practice, this study recommends that efforts need to focus on standardizing SUP protocols, implementing pharmacist-led stewardship programs, and reinforcing the role of enteral nutrition as a protective strategy. Furthermore, targeted educational initiatives and awareness campaigns are recommended to promote evidence-based prescribing practices among critical care providers. In addition, integrating computerized physician order entry (CPOE) systems with embedded clinical decision support tools can help reduce reliance on individualized decision-making and ensure adherence to appropriate SUP indications.

## Conclusions

This study highlights a high level of awareness of SUP guidelines among ICU clinicians but reveals considerable variability in adherence and practice patterns. Despite evidence supporting selective use for high-risk patients, many clinicians continue to initiate and prolong PPI use in all ICU patients, potentially increasing the risk of *Clostridium difficile* and other nosocomial infections. While recent findings question the mortality benefit of PPIs, their use in selected high-risk patients remains supported due to their role in reducing clinically significant gastrointestinal bleeding. We recommend implementing standardized SUP protocols, pharmacist-led stewardship programs, and reinforcing early enteral nutrition as a non-pharmacologic strategy. Electronic decision-support tools such as computerized physician order entry with embedded checklists may also improve appropriate initiation and timely discontinuation. Future research should evaluate the clinical outcomes and effectiveness of these interventions, including patient-level outcomes such as gastrointestinal bleeding and *C. difficile* infection.

## Appendices

Research questionnaire

Age: ----- Gender: □ Male □ Female Nationality: -----

This questionnaire is used to collect data that will investigate your knowledge and subsequently practice regarding Stress Ulcer Prophylaxis in patients at Intensive Care Unit. This questionnaire will solely be used for research purposes only, your response will be anonymous and will never be linked to your personality.

I AGREE to participate voluntarily in this questionnaire and I know its purpose.

□YES  □NO

Signature:

Which department do you work at? □ Anesthesiology □ General internal medicine □ Critical care medicine □ Emergency medicine □ General surgery

What type of practice do you work in? □ General Government hospital □ Military hospital □ Tertiary specialized hospital

What is the size of your ICU unit? □ <20 □ 10-20 □ >20

What types of patients do you care for in your ICU? (You can choose >1) □ Medical □ Surgical □ Trauma □ Neuro □ Cardiac surgery □ Burns

How many years of overall medical experience do you have? (Including your ICU practice) □ 0-3 years □ 3-6 years □ 6-10 years □ >10 years

What is the duration of your ICU experience? □ < 4 months □ 6-12 months □ 1-5 years □ >5 years

Are you aware of any guidelines regarding stress ulcer prophylaxis? □ Yes □ No

If yes, at what year was the guideline you are aware of released? □ Before 2010 □ 2010-2015 □ 2015-2020 □ After 2020

Is there any SUP protocol being followed in your ICU? □ Yes □ No

(If No, please ignore the next question)

If yes, who is responsible for following compliance of SUP? □ Attending consultant □ Caring ICU physician □ The primary nurse □ Clinical pharmacist

In your opinion, should all patients admitted to ICU receive SUP? □ Yes □ No

In your opinion, is the incidence of all types of bleeding from stress ulceration uncommon in ICU patients? □ Yes □ No

In your opinion, could the risk of GI bleeding be altered by the use of acid suppressive therapy? □ Yes □ No

In your opinion, is early enteral tube feeding (initiated within 48 hours of ICU admission) protective against stress ulceration? □ Yes □ No

In your opinion, only patients with risk factors for stress-related mucosal diseases should receive SUP? □ Yes □ No

When is the best time to start SUP? □ Upon arrival to hospital □ At the time of arrival to ICU □ Only if there is a specific indication

What are the independent predictors of clinically important GI bleeding in ICU? (You can choose >1) □ Coagulopathy □ NSAIDS □ DM □ Mechanical ventilation >48 hours

Which one of the following conditions is considered coagulopathy that requires SUP? (You can choose >1) □ Platelet count < 50000 □ Platelet count < 100000 □ INR > 1.5 □ INR > 1.2

When do you consider initiating SUP in mechanically ventilated patients? □ At the time of initiation of ventilation □ Mechanical ventilation >24 hours □ Mechanical ventilation >48 hours □ Mechanical ventilation >7 days

Which of the following are considered minor risk factors for GI bleeding from stress ulcer? (You can choose >1) □ Chronic hepatic failure □ Glucocorticoid therapy (≥ 250mg hydrocortisone equivalent per day) □ Coagulopathy □ Acute or chronic renal failure □ Respiratory failure

The presence of how many minor risk factors warrants use of SUP prophylaxis? □ 1 □ 2 □ 3 □ 4 □ ≥5

SUP should be initiated in the ICU setting if? □ At least 1 independent/major risk factor is present □ When at least 1 minor risk factor is present □ Even when there are no risk factors

Which one of the following is the SUP of choice in presence of major risk factors? □ IV PPI □ Enteral PPI □ Sucralfate

Which one of the following is the best SUP to prevent gastric bleed? □ IV PPI □ Enteral PPI □ Sucralfate

Which one of the following modalities of SUP has maximum risk of ventilator-associated pneumonia? □ PPI □ Sucralfate

Which one of the following is an adverse effect of PPIs? □ VAP □ Clostridium difficile infection □ MI □ Dementia

Once initiated in an ICU patient, SUP should? □ Continue throughout the patient’s ICU stay □ Continue throughout the patient’s hospital stay □ Should be continued for life □ Should be discontinued when there is no remaining indication

What is the current approach to SUP in your ICU? (You can choose >1) □ Each ICU physician can decide on case-by-case basis □ An informal unwritten ICU policy determines practice □ Standardized paper-based pre-printed physician’s order □ Standardized orders by computerized physician order entry □ Other: _____

In your opinion, which of the following can lead to lack of adherence to SUP? (You can choose >1) □ Overwhelming patient load □ Lack of daily medication review □ Lack of clinical pharmacist presence □ Lack of knowledge
